# Direct Effects of (−)-Epicatechin and Procyanidin B2 on the Respiration of Rat Heart Mitochondria

**DOI:** 10.1155/2015/232836

**Published:** 2015-02-24

**Authors:** Dalia M. Kopustinskiene, Arunas Savickas, David Vetchý, Ruta Masteikova, Arturas Kasauskas, Jurga Bernatoniene

**Affiliations:** ^1^Department of Pharmaceutical Technology and Social Pharmacy, Medical Academy, Lithuanian University of Health Sciences, Eiveniu 4, LT-50161 Kaunas, Lithuania; ^2^Department of Pharmaceutics, University of Veterinary and Pharmaceutical Sciences Brno, Palackeho 1/3, 612 42 Brno, Czech Republic; ^3^Department of Biochemistry, Medical Academy, Lithuanian University of Health Sciences, Kaunas, A. Mickeviciaus 9, LT-44307 Kaunas, Lithuania

## Abstract

Flavonol (−)-epicatechin and its derived dimer procyanidin B2, present in high amounts in cocoa products, have been shown to exert beneficial effects on the heart and cardiovascular system; however, their mechanism of action has not been fully elucidated. We studied effects of (−)-epicatechin and procyanidin B2 on the oxidative phosphorylation of isolated rat heart mitochondria. (−)-Epicatechin and procyanidin B2 had stimulating effect (up to 30% compared to control) on substrate-driven (State 2) mitochondrial respiration. Their effect was dependent on the respiratory substrates used. (−)-Epicatechin at higher concentrations (from 0.27 *µ*g/mL) significantly decreased (up to 15%) substrate- and ADP-driven (State 3) mitochondrial respiration in case of pyruvate and malate oxidation only. Procyanidin B2 (0.7–17.9 ng/mL) inhibited State 3 respiration rate up to 19%, the most profound effect being expressed with succinate as the substrate. (−)-Epicatechin at concentrations of 0.23 *µ*g/mL and 0.46 *µ*g/mL prevented loss of the cytochrome *c* from mitochondria when substrate was succinate, supporting the evidence of membrane stabilizing properties of this flavonol. Thus, both (−)-epicatechin and procyanidin B2 directly influenced mitochondrial functions and the observed effects could help to explain cardiometabolic risk reduction ascribed to the consumption of modest amounts of cocoa products.

## 1. Introduction

(−)-Epicatechin is a compound representative of the flavanols—a subfamily of the polyphenolic compounds—flavonoids, abundant in grape seeds, grape skin, tea, cola nuts, strawberries, and red wine [1]. Furthermore, (−)-epicatechin together with its most common dimmer procyanidin B2 are the main biologically active compounds present in cocoa products including chocolate [2].

The molecular actions of flavanols and procyanidins largely depend on their bioavailability. Total plasma concentrations of (−)-epicatechin and its metabolites were found in the low micromolar range after 1 h after ingestion of flavanol containing foods [3, 4]. However, the estimated bioavailability of procyanidin B2 is in lower nanomolar range [4].

There is accumulating evidence that (−)-epicatechin and its derivatives have significant role in prevention of cardiovascular diseases in humans [5]. Potent antioxidant action, modulation of cell signalling, stabilization of membranes, improvement of endothelial function, reduction of the blood pressure, and protection of mitochondria, main organelles responsible for cellular energy supply, are being proposed as possible mechanisms of beneficial effects of (−)-epicatechin [4].

Meanwhile, procyanidins and especially procyanidin B2 have been demonstrated to exert chemopreventive action, a relatively new and promising strategy to prevent cancer [6], as well as modulation of signal transduction pathway, anti-inflammatory properties [7], and antioxidant and prooxidant activity [8].

Coronary artery disease, and its serious outcome acute myocardial infarction, is one of the major causes of morbidity and mortality in the world. In the cardiomyocytes, high levels of reactive oxygen and nitrogen species, generated by mitochondria, lead to contractile dysfunction and the cell death (for recent review, see [9–11]). However, the direct effects of (−)-epicatechin and procyanidin B2 on cardiac mitochondria have not been elucidated yet; therefore, the aim of our study was to study the influence of both compounds on the oxidative phosphorylation in heart mitochondria respiring on different substrates: pyruvate and malate, succinate (in the presence of amytal), and palmitoyl-L-carnitine + malate.

## 2. Materials and Methods

### 2.1. Chemicals and Animals

(−)-Epicatechin (purity ≥ 98%), procyanidin B2 (purity ≥ 90%), and all other chemicals used in this work were from Sigma-Aldrich (St. Louis, MO, USA). (−)-Epicatechin and procyanidin B2 were used dissolved in ethanol. Solvent controls were run in all tests and had no effect on the evaluated functions. Male Wistar rats (age ~3 months), weighing 250–300 g, were used for the study.

### 2.2. Isolation of Rat Heart Mitochondria

According to the requirements of the EU Directive 2010/63/EU for animal experiments the study protocol was approved by the Lithuanian Animal Ethics Committee (SFVS License no. 0155). Rats were killed by an increasing concentration of CO_2_ in the air followed by cervical dislocation. Hearts of rats were excised and rinsed in ice-cold 0.9% KCl solution. Heart mitochondria were isolated in the medium containing 220 mM mannitol, 70 mM sucrose, 5 mM N-tris[Hydroxymethyl]methyl-2-aminoethane-sulfonic acid, 0.5 mM EGTA (pH 7.4, adjusted with Trisma base; 2°C), and 2 mg/mL bovine serum albumin (BSA; fraction V, A4503, Sigma). The homogenate was centrifuged at 750 ×g for 5 min, then the supernatant was recentrifuged at 10,000 ×g for 10 min, and the pellet was washed once (10 min 10,000 ×g) in the isolation medium without BSA, suspended in it and kept on ice. The mitochondrial protein concentration was determined by applying the biuret method with BSA used as standard.

### 2.3. Registration of Mitochondrial Respiration Rate

Oxygen uptake rate was recorded at 37°C by means of the Clark-type electrode system in a solution containing 20 mM imidazole, 20 mM taurine, 0.5 mM dithiothreitol, 1.6 mM MgCl_2_, 100 mM MES, 3 mM KH_2_PO_4_, 3 mM CaK_2_EGTA, and 7.1 mM K_2_EGTA (free Ca^2+^ concentration: 0.1 *μ*M) (pH 7.1 adjusted with KOH at 37°C) with different substrates: 6 mM pyruvate and 6 mM malate or 12 mM succinate (plus 2 mM amytal) or 9 *μ*M palmitoyl-L-carnitine and 0.24 mM malate as substrates. The solubility of oxygen was estimated to be 422 nmol O/mL [12]. Mitochondrial respiration rate was expressed as nmol O/min/mg protein. The final mitochondrial protein concentration in all experiments was 0.5 mg/mL. Mitochondrial State 2 respiration rate was registered after addition of mitochondria and substrates. Later, after addition of 1 mM of ADP, the maximal State 3 respiration rate (V_ADP_) was measured. State 3 respiration rate reflects the maximal capacity of mitochondria to synthesize ATP. The tested compounds were added after registration of the State 2 or State 3 respiration. The intactness of the outer mitochondrial membrane was assessed based on the stimulation of the State 3 respiration rate by the addition of exogenous cytochrome *c* (32 *μ*M).

### 2.4. Statistical Analysis

Data are presented as mean ± SEM. Statistical analysis was performed by one-way analysis of variance (ANOVA), followed by Dunnett's post hoc test using the software package Prism v. 5.04 (GraphPad Software Inc., La Jolla, CA, USA). A value of *P* < 0.05 was taken as the level of significance.

## 3. Results

**Figure 1:**
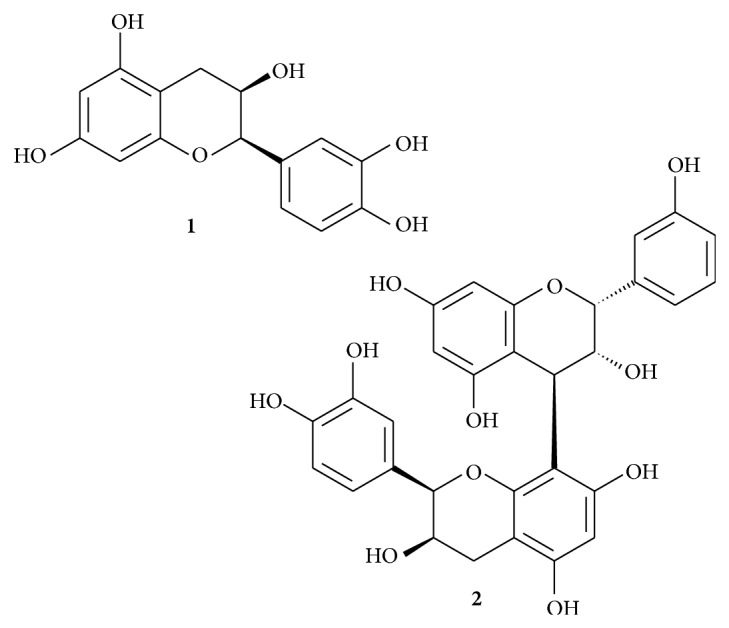
Chemical structure of (−)-epicatechin (**1**) and procyanidin B2 (**2**).

We investigated effects of (−)-epicatechin and its derived dimer procyanidin B2 (Figure 1) on the respiration rate of isolated rat heart mitochondria, oxidizing NAD-dependent substrates pyruvate and malate, FAD-dependent substrate succinate (in the presence of amytal), and the main respiratory substrate of heart mitochondria, fatty acid derivative palmitoyl-L-carnitine. In our study, mitochondrial functions were evaluated by recording mitochondrial respiration rate in the State 2 (substrate oxidation), State 3 (substrates + 1 mmol/L ADP, V_ADP_), and State 3 in the presence of exogenous cytochrome *c* (32 *μ*mol/L, V_ADP+Cyt  *c*_). The concentration range of tested compounds was chosen to be in lower micromolar range for (−)-epicatechin and lower nanomolar range for procyanidin B2 according to their bioavailability data [3, 4].

### 3.1. Influence of (−)-Epicatechin and Procyanidin B2 on the Substrate-Driven (State 2) Mitochondrial Respiration

**Figure 2:**
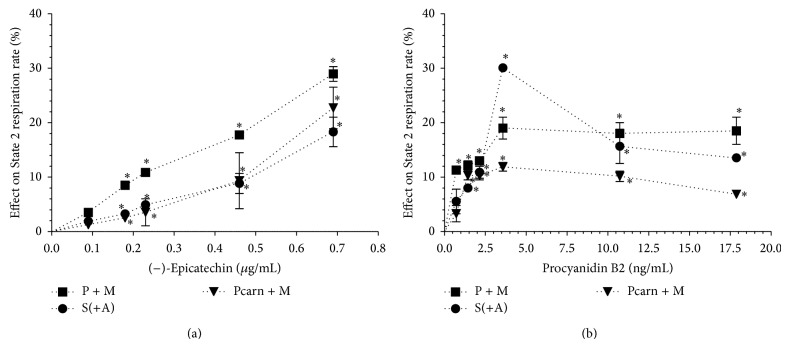
Effect of (−)-epicatechin (a) and procyanidin B2 (b) on the respiration rate of isolated rat heart mitochondria in the State 2. Effect was expressed in % of initial respiration rate, which was 76 ± 2 nmol O/min/mg protein (substrates, pyruvate and malate, P + M), 200 ± 14 nmol O/min/mg protein (substrate succinate in the presence of amytal, S(+A)), and 89 ± 6 nmol O/min/mg protein (substrates, palmitoyl-L-carnitine and malate, Pcarn + M). ^*^ *P* < 0.05 versus control, *n* = 5. The results were analysed with one-way analysis of variance (ANOVA) followed by Dunnett's post hoc test.

Our results showed that (−)-epicatechin starting from 0.18 *μ*g/mL in a concentration-dependent manner significantly increased the State 2 respiration rate of isolated rat heart mitochondria with all tested substrates (Figure 2(a)). The stimulating effect of (−)-epicatechin was dependent on the respiratory substrates used (up to 30% with pyruvate and malate, up to 23% with palmitoyl-L-carnitine, and up to 18% with succinate).

Procyanidin B2 also increased State 2 respiration rate of isolated rat heart mitochondria starting from 0.7 ng/mL with all tested substrates; however, the stimulating pattern differed from (−)-epicatechin (Figure 2(b)). We observed the peak stimulating effect at 3.6 ng/mL which was also dependent on the respiratory substrates used (30% with succinate, 19% with pyruvate, and malate and 12% with palmitoyl-L-carnitine). However, procyanidin B2 at higher concentrations, 11 and 18 ng/mL, increased mitochondrial respiration in State 2 at lower extent than at 3.6 ng/mL.

### 3.2. Effects of (−)-Epicatechin and Procyanidin B2 on the Substrate- and ADP-Driven (State 3) Mitochondrial Respiration

**Figure 3:**
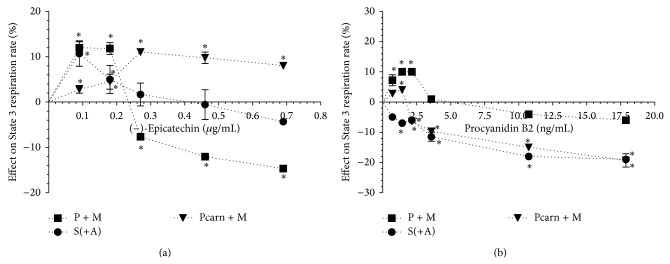
Effect of (−)-epicatechin (a) and procyanidin B2 (b) on the respiration rate of isolated rat heart mitochondria in State 3. Effect was expressed in % of substrates- and ADP-driven respiration rate, which was 455 ± 16 nmol O/min/mg protein (substrates, pyruvate and malate, P + M), 453 ± 7 nmol O/min/mg protein (substrate succinate in the presence of amytal, S(+A)), and 392 ± 10 nmol O/min/mg protein (substrates, palmitoyl-L-carnitine and malate, Pcarn + M). ^*^ *P* < 0.05 versus control, *n* = 5. The results were analysed with one-way analysis of variance (ANOVA) followed by Dunnett's post hoc test.

Our results showed that (−)-epicatechin at lower concentrations (starting from 0.09 *μ*g/mL) stimulated up to 10% mitochondrial respiration rate in State 3 (Figure 3(a)). The peak stimulating effect was observed at 0.18 *μ*g/mL of (−)-epicatechin with pyruvate and malate, at 0.09 *μ*g/mL with succinate in the presence of amytal, and at 0.27 *μ*g/mL with palmitoyl-L-carnitine as substrates. At higher concentrations the effect of (−)-epicatechin on State 3 mitochondrial respiration was dependent on the respiratory substrates used. In the case of pyruvate and malate oxidation, (−)-epicatechin starting from 0.27 *μ*g/mL significantly inhibited mitochondrial respiration rate in a concentration-dependent manner up to 15% at 0.7 *μ*g/mL. However, in the case of palmitoyl-L-carnitine oxidation, the stimulatory effect of (−)-epicatechin decreased at higher concentrations, although remaining at the level of 8% at 0.7 *μ*g/mL, the highest concentration tested. In the case of succinate (in the presence of amytal) oxidation, (−)-epicatechin from 0.27 *μ*g/mL had no influence on State 3 respiration rate.

Procyanidin B2 (0.7–17.9 ng/mL) significantly inhibited State 3 respiration rate up to 19% with succinate (in the presence of amytal) as the substrate (Figure 3(b)). In the case of pyruvate and malate as well as palmitoyl-L-carnitine oxidation, procyanidin B2 at lower concentrations (0.7–2.1 ng/mL and 1.4 ng/mL) had mild stimulatory effect (10% and 4%, resp.) on the mitochondrial respiration rate in State 3. However, at higher concentrations (Figure 3(b)) the inhibitory effect of procyanidin B2 was observed, lesser with pyruvate and malate as substrates (up to 6% compared to that up to 19% with other substrates tested).

### 3.3. Effects of (−)-Epicatechin and Procyanidin B2 on Cytochrome *c* Release from Rat Heart Mitochondria

**Figure 4:**
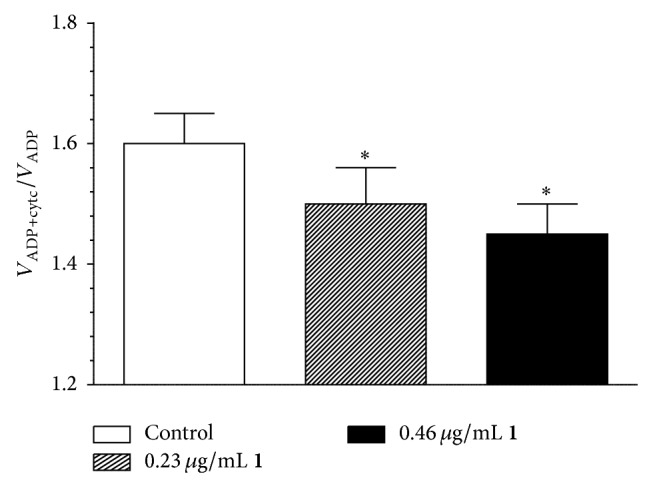
Effect of (−)-epicatechin (**1**) on the cytochrome *c* release from isolated rat heart mitochondria oxidizing succinate in the presence of amytal. V_ADP+Cyt  *c*_, cytochrome *c* (32 *μ*M), stimulated State 3 respiration rate of heart mitochondria, V_ADP_, State 3 respiration rate of heart mitochondria. ^*^ *P* < 0.05 versus control, *n* = 5. The results were analysed with one-way analysis of variance (ANOVA) followed by Dunnett's post hoc test.

Cytochrome *c* is a 12 kDa protein, loosely bound to the outer surface of the inner mitochondrial membrane and functioning as a single electron carrier from the cytochrome *bc*_1_ complex to cytochromoxidase in the final step of the electron transport chain [13]. Since intact mitochondrial outer membrane is not permeable to cytochrome *c*, its integrity is evaluated based on the degree of mitochondrial State 3 (substrates + ADP-driven) respiration stimulation by exogenous cytochrome *c* (V_ADP+Cyt  *c*_/V_ADP_). The release of cytochrome *c* is more pronounced when mitochondria oxidize FAD-dependent substrate and less pronounced while oxidizing NAD-dependent substrates. Since under our experimental conditions the release of cytochrome *c* from mitochondria oxidizing pyruvate and malate or palmitoyl-L-carnitine was negligible, we tested effects of (−)-epicatechin and procyanidin B2 on cytochrome *c* release from rat heart mitochondria oxidizing succinate. Our results have demonstrated that (−)-epicatechin (Figure 4) but not procyanidin B2 (data not shown) decreased cytochrome *c* release from mitochondria when substrate was succinate (in the presence of amytal), supporting the evidence of membrane stabilizing properties of this flavonol [4, 14].

## 4. Discussion

(−)-Epicatechin and its derivatives were shown to exert protective effects in various models of metabolic stress and cardiovascular diseases [4]. Pretreatment of cells with (−)-epicatechin minimized subsequent stroke injury during brain ischemia [15], protected PC12 cells from amyloid beta protein-induced neurotoxicity [16]. Furthermore, (−)-epicatechin was suggested as a potential remedy to treat nociceptive hypersensitivity in diabetic patients [17] and conditions associated with tissue oxidative stress [18], and it was reported to have insulinogenic and insulin-like activities [19].

Procyanidin B2 protected cardiomyocytes exposed to ischemia/reperfusion [20]. It was shown to exert both antioxidant and prooxidant properties [8] and anti-inflammatory activity [7]. Furthermore, procyanidin B2 was cytotoxic to MCF-7 human breast cancer cells and has been suggested to be tested further as potential antineoplastic agent [6].

Mitochondria are the main sites of cellular energy supply. Therefore, modulation of their functional activity may be very important for preserving cell viability under normal conditions and during metabolic stress. Impaired mitochondrial function represents an early manifestation of endothelial dysfunction and contributes to the development of cardiovascular diseases [21]. The consumption of modest amounts of cocoa products is associated with ~40% reduction in cardiometabolic risk [22]. The beneficial effect of cocoa has been ascribed to (−)-epicatechin and its derivatives [3]. Furthermore, several recent studies indicate that protective action of these compounds occurs by interactions with intracellular targets, including mitochondria [21, 23, 24].

In State 2 mitochondrial respiration rate mainly depends on the passive proton flux through the mitochondrial inner membrane [25]. Thus, our results show that both (−)-epicatechin and procyanidin B2 possess a concentration-dependent uncoupling effect on oxidative phosphorylation in cardiac mitochondria (Figure 2). Based on the principles of linear nonequilibrium thermodynamics, it was proposed that the optimal efficiency of oxidative phosphorylation could be reached when mitochondria are slightly uncoupled [26]. Furthermore, uncoupling can prevent ROS formation in mitochondria [27]. As there is a growing evidence that a mild uncoupling is a very important mechanism of cardioprotection (for recent reviews, see [28, 29]), our results imply that both (−)-epicatechin and procyanidin B2 could be beneficial in the prevention of cardiovascular diseases and metabolic stress.

In State 3 complex I-dependent substrates-supported mitochondrial respiration rate depends both on the activity of mitochondrial respiratory chain and phosphorylation [30, 31], whereas, in the case of succinate oxidation, ATP synthesis, transport, and hydrolysis are responsible only for ~10% of the respiration rate [32]. Thus, our results imply that (−)-epicatechin slightly suppresses pyruvate and malate oxidation and stimulates fatty acid oxidation. Procyanidin B2 mildly inhibited succinate dehydrogenase and fatty acid oxidation, although at lower concentrations both (−)-epicatechin and procyanidin B2 stimulated phosphorylation (i.e., ATP production) in heart mitochondria. The latter effect was confirmed based on uncoupled mitochondrial respiration rate measurements (data not shown). The stimulation of respiration could be observed only in State 3 mitochondrial respiration, thus excluding possibility of effect on respiratory chain and confirming the effect on ATP production.

Mitochondrial respiratory inhibition-triggered ROS signaling [33] promotes preconditioning-like cardioprotection in the heart; therefore, our data suggest that (−)-epicatechin and procyanidin B2 could also indirectly decrease ROS production. This is in line with other investigations where (−)-epicatechin and its derivatives were shown to play an important role in the ROS scavenging [4]. Consumption of (−)-epicatechin rich food was associated with improvement in endothelial function and with reduction in blood pressure [2] due to augmentation of the level of NO in endothelial cells [34]. Moreover, (−)-epicatechin and its derivatives enhanced mitochondrial function and protein levels in cultured bovine coronary artery endothelial cells [21]. Increased mitochondrial respiration and ROS production during State 3 respiration, increased rigidity of mitochondrial membranes, and resistance to calcium-induced mitochondrial swelling were demonstrated after oral treatment of mice with (−)-epicatechin (1 mg/kg) for 10 days [23]; however, the authors did not test direct effects of (−)-epicatechin incubation on isolated mitochondria. The preventive effects of (−)-epicatechin on oxidative stress, cardiac mitochondrial damage, altered membrane bound adenosine triphosphatases, and minerals were reported in isoproterenol-induced myocardial infarction model [14, 24]. Furthermore, (−)-epicatechin induced phosphorylation/activation of endothelial nitric-oxide synthase, protein kinase B, and heat shock protein 90 [35]. Thus, there might be other cellular pathways involved in the protective effect of (−)-epicatechin, including heat shock response and cellular antioxidant systems, that is, glutathione peroxidases and transferases.

(−)-Epicatechin was suggested not only as a novel cardioprotective compound but also as an emerging option to treat disorders associated with mitochondrial dysfunction [36]. Furthermore, the protection from cardiovascular diseases, myocardial infarction, ischemia [20, 37], and cancer that is associated with a high consumption of fruit and vegetables could be in part explained by the capacity of flavanols and related procyanidins to modulate proinflammatory and oncogenic signals [4].

## 5. Conclusions

In line with these overviewed reports, for the first time, to our knowledge, our results demonstrate that (−)-epicatechin and procyanidin B2 directly influence mitochondrial functions. The observed uncoupling of oxidation from phosphorylation, stimulation of phosphorylation at lower concentrations, and inhibition of respiratory chain at higher concentrations as well as (−)-epicatechin-reduced release of cytochrome *c* from mitochondria might be responsible for the beneficial cardioprotective effects associated with the consumption of modest amount of cocoa products, rich in (−)-epicatechin and its derivatives.
